# Complete Blood Count-Derived Indices as Prognostic Factors of 5-Year Outcomes in Patients With Confirmed Coronary Microvascular Spasm

**DOI:** 10.3389/fcvm.2022.933374

**Published:** 2022-06-30

**Authors:** Jacek Bil, Natalia Pietraszek, Robert J. Gil, Leszek Gromadziński, Dariusz Onichimowski, Rakesh Jalali, Adam Kern

**Affiliations:** ^1^Department of Invasive Cardiology, Center of Postgraduate Medical Education, Warsaw, Poland; ^2^Department of Invasive Cardiology, Central Clinical Hospital of the Ministry of Interior and Administration, Warsaw, Poland; ^3^Department of Cardiology and Internal Medicine, School of Medicine, Collegium Medicum, University of Warmia and Mazury in Olsztyn, Olsztyn, Poland; ^4^Department of Anesthesiology and Intensive Care, School of Medicine, Collegium Medicum, University of Warmia and Mazury, Olsztyn, Poland; ^5^Clinical Department of Anesthesiology and Intensive Care, Regional Specialist Hospital in Olsztyn, Olsztyn, Poland; ^6^Emergency Medicine Department, School of Medicine, Collegium Medicum, University of Warmia and Mazury in Olsztyn, Olsztyn, Poland; ^7^Clinical Emergency Department, Regional Specialist Hospital in Olsztyn, Olsztyn, Poland; ^8^Department of Cardiology, Regional Specialist Hospital in Olsztyn, Olsztyn, Poland

**Keywords:** RDW, PDW, MPV, acetylcholine test, coronary microcirculation

## Abstract

**Background:**

Coronary microcirculatory dysfunction is a meaningful factor in the development of ischemic heart disease. We investigated the relationship between coronary microvascular spasm and complete blood count indices.

**Methods:**

Between 2010 and 2013, we performed acetylcholine test (AChT) in subjects with suspicion of angina evoked by epicardial coronary spasm or coronary microvascular spasm according to COVADIS criteria. We administered acetylcholine in increasing doses of 25, 50, and 75 μg into the right coronary artery and 25, 50, and 100 μg into the left coronary artery. Patients were followed up for 60 months.

**Results:**

In total, 211 patients (60.5 ± 7.8 years, 67.8% women) were included in the study. The AChT revealed angina due to epicardial coronary spasm in 99 patients (46.9%) and coronary microvascular spasm in 72 (34.1%). White blood cell (WBC), red blood cell distribution width (RDW), platelets (PLT), mean platelet volume (MPV), and platelet distribution width (PDW) values were significantly higher in patients with coronary microvascular spasm than in patients from the other two groups, i.e., epicardial coronary spasm and negative AChT. PDW showed the highest sensitivity (65%) and specificity (72%) at the cutoff value of 15.32% [area under the curve, 0.723; 95% confidence interval (CI) 0.64–0.83; *P* < 0.001]. Independent risk factors for coronary microvascular spasm diagnosis using AChT were as follows: female sex (OR, 1.199), PDW (OR, 2.891), and RDW (OR, 1.567).

**Conclusion:**

PDW and RDW are significantly associated with the diagnosis of coronary microvascular spasm in patients undergoing AChT as well as with poor prognosis in such patients at 5 years.

## Introduction

Coronary microcirculatory dysfunction is a meaningful factor in the development of ischemic heart disease (IHD). The long-term prognosis of coronary microcirculatory dysfunction is often thought to be relatively benign. Nevertheless, standard medical management including vasodilators is often ineffective and sometimes cannot prevent myocardial infarction (MI) with non-obstructive coronary arteries (MINOCA) or fatal arrhythmias (1–3).

Although angiographic provocation testing for epicardial coronary spasm or coronary microcirculatory dysfunction is available, still many clinicians concentrate only on atherosclerotic stenoses, with less emphasis on other potential causes like coronary spasm. Therefore, it is vital to raise more awareness on epicardial coronary spasm or coronary microcirculatory dysfunction in clinicians, but also to identify reliable markers for screening patients who may be candidates for a more proactive clinical investigation embracing provocative acetylcholine test (AChT) (4–6).

Interestingly, the red blood cell (RDW) and platelet (PDW) distribution widths reportedly are strong predictors of the frequency as well as the outcomes of various cardiovascular diseases (CVD) (7–10). The mechanism for the links between increased RDW or PDW values and the CVD prognosis remain unclear. Nevertheless, recent studies indicated that an interplay between endothelial dysfunction, chronic inflammatory response as well as oxidative stress might explain this association (2, 3, 11).

In the literature not many studies have assessed this issue, such as those evaluating RDW’s predictor role in cardiac syndrome X (12), RDW in vasospastic angina (13), RDW and plateletcrit (Pct) in slow flow phenomenon assessment (14), and neutrophil-to-lymphocyte ratio (NLR) and index of microcirculatory resistance in patients with ST-segment elevation MI undergoing primary percutaneous coronary intervention (15). To our knowledge, there is no data on the association of complete blood count indices with microvascular spasm evaluated in coronary invasive provocative tests or in MINOCA patients. Therefore, we assessed complete blood count indices as potential markers for long-term outcomes in patients with coronary microvascular spasm. We compared systemic inflammatory markers such as NLR and platelet-to-lymphocyte ratio (PLR) as well as various red blood cells and platelet (PLT) indices such as RDW, mean PLT volume (MPV), PDW, and Pct.

## Materials and Methods

### Study Population and Study Plan

**FIGURE 1:**
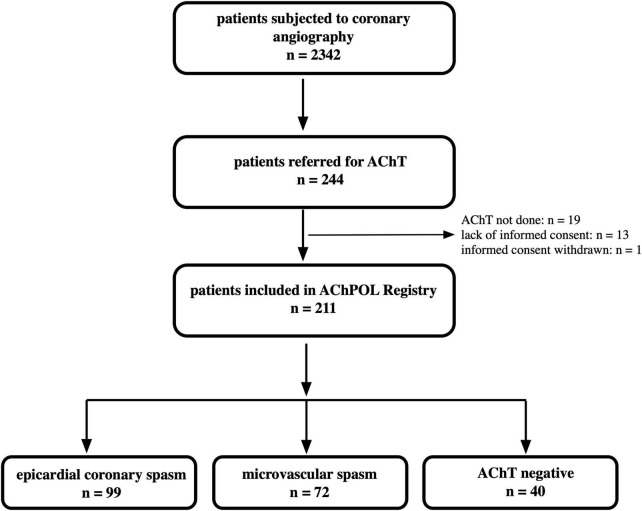
The study flowchart. AChT, acetylcholine test.

It was a prospective observational study. We included patients enrolled to the AChPOL Registry between December 2010 and March 2013 (Figure 1) (16). We performed AChT in patients who underwent diagnostic coronary angiography, had non-obstructive coronary arteries (no epicardial stenosis ≥ 50%), and were referred for further investigation due to suspicion of angina evoked by epicardial coronary spasm or coronary microcirculatory dysfunction according to Coronary Vasomotion Disorders International Study Group (COVADIS) criteria (17). The exclusion criteria were as follows: (1) severe chronic obstructive pulmonary disease, (2) chronic kidney disease with serum creatinine > 2.0 mg/dL, (3) observed spontaneous spasm, (4) PLTs were < 100,000/μL, (5) active malignancy or (6) active infection.

The institutional review board approved the registry protocol, and all patients provided written informed consent before enrollment to the AChPOL Registry. Study reporting conformed to the Strengthening the Reporting of Observational Studies in Epidemiology (STROBE) statement along with references to the STROBE statement and the broader Enhancing the QUAlity and Transparency Of health Research (EQUATOR) guidelines (18).

### Interventional Procedure and Concomitant Medications

All patients underwent intracoronary provocation with acetylcholine (ACh) according to a standardized protocol. We administered increasing ACh doses over a period of 3 min into the coronary arteries *via* a diagnostic catheter [25, 50, and 100 μg for the left coronary artery (LCA), 25, 50, and 75 μg for the right coronary artery (RCA)] (17, 19, 20). We judged the AChT as positive for epicardial coronary spasm when we observed focal or diffuse reduction of epicardial coronary diameter ≥ 90% comparing to baseline view (following intracoronary nitroglycerine infusion) together with evoking patient’s symptoms and ischemic electrocardiographic (ECG) changes. We also recorded the location and type of epicardial coronary spasm. Focal spasm was defined as vessel narrowing within one isolated or two adjacent coronary segments according to the segment definition of the American Heart Association. Diffuse spasm was recognized when present in ≥ two neighboring coronary segments. Coronary microvascular spasm was recognized when typical ischemic ST-segment changes and angina developed with epicardial coronary spasm < 90% in diameter reduction. Patients with no angina, spasm, or ST-segment changes were judged to have a negative AChT response (normal coronary vasoreactivity) (17, 21, 22). In coronary arteries with stenoses of > 40% the fractional flow reserve was done, mostly during the initial coronary angiography (20).

### Clinical Follow-Up and Endpoints

All patients were discharged on optimal medical treatment, including calcium channel blockers (CCB) uptitrated at the highest tolerated doses. Adverse events were recorded throughout the follow-up period. Follow-up was performed at 12, 24, 36, 48, and 60 months by telephone and/or at clinical visits.

We assessed the rates of death from any cause, cardiac death, recurrent acute coronary syndrome (ACS), and recurrent angina requiring hospitalization. Cardiac death was defined as death from an acute MI, sudden cardiac death, and death due to heart failure and cardiac procedures. All death cases were deemed cardiac unless proven otherwise. MI was defined according to the third universal definition (23).

### Laboratory Tests

For complete blood count measurement, venous blood samples were collected in K2-ethylenediaminetetraacetic acid (EDTA) tubes at admission before performing catheterization and AChT. The neutrophil, lymphocyte, PLT, MPV, PDW, and RDW values were analyzed on an automated hematologic analyzer (Sysmex Corporation, Kobe, Japan) within 60 min after sample collection. The laboratory reference values were as follows: PLT 150 to 400 × 10^9^/L; MPV 9.4–12.4 fL; PDW 9.0–17.0 fL, RDW 11.5%–15%, NLR 1.2–4, and PLR 75–199.

### Statistical Analysis

We present the data as means [standard deviation (SD)] or median [interquartile range (IQR)] or percentage. We used the χ^2^ or Fisher’s exact test in all categorical variables, while one-way analysis of variance or the Kruskal–Wallis *H* test was used for all continuous variables. *Post hoc* analyses using 2-tailed Tukey’s honestly significant difference test were conducted to verify the differences between the groups. No corrections for multiple comparisons were applied.

In the next step, univariable and multivariable logistic regression analyses were conducted to evaluate the impact of potential risk factor on odds of microvascular spasm diagnosis. The stepwise backward regression with AIC minimization procedure was used on full multivariable model in variables selection for reduced multivariable model. Then, we used receiver-operating characteristic (ROC) curves to assess the diagnostic values of analyzed parameters.

Finally, the time to event data was investigated with the Kaplan–Meier estimator of survival curve, and a log-rank test was applied to evaluate the survival distributions between groups according to AChT results. To identify independent predictors of MI/chest pain hospitalizations in coronary microvascular spasm group at 5 years we used Cox proportional hazard regression models. Demographic, clinical and laboratory parameters that significantly differed between subgroups according to the AChT result were included into the model.

Level of statistical significance was set as 0.05. Two-sided tests were applied. We performed statistical analyses with R 3.0.2 for OS (R Foundation, Vienna, Austria). As stated previously, no formal sample size calculation was performed, as the study had explorative character and the patients’ number was restricted by the number of patients referred for the AChT and length of enrollment period (16).

## Results

The enrollment period was from December 2010 to March 2013. We analyzed 211 patients [mean age 60.5 ± 7.8 years, 143 women (67.8%)] who underwent the AChT and for whom all required laboratory tests were available. Relatively high hypertension (62.6%, *n* = 132) and dyslipidemia (45.5%, *n* = 96) rates were observed. Table 1 presents the detailed characteristics. The AChT revealed angina due to epicardial coronary spasm in 99 patients (46.9%) and coronary microvascular spasm in 72 (34.1%). In 40 (18.9%) patients, the AChT was negative (no symptoms, no ECG changes, no epicardial spasm, no increased blush).

**TABLE 1:** Baseline clinical characteristics.

Parameter	Microvascular spasm N = 72	Epicardial coronary spasm N = 99	Negative AChT N = 40	P
Age [years]	58.4 ± 8.9 ^	59 ± 9.6 ^	68.1 ± 10.8	0.02
Females	60 (83.3) ^	67 (67.7) ^	16 (40)	0.03
**Angina:**				
Exertional	53 (73.6)	30 (30.3) ^ ^,&^	29 (72.5)	0.01
At rest	44 (61.1) ^	71 (71.7) ^	16 (40)	0.02
At night	39 (54.2) ^	62 (62.6) ^	9 (22.5)	0.01
Arterial hypertension	51 (70.8) ^	62 (62.6)	19 (47.5)	0.04
Diabetes type 2	4 (5.6) ^	5 (5.1) ^	7 (17.5)	0.04
Dyslipidemia	46 (63.9) ^ ^,&^	35 (35.4)	15 (37.5)	0.01
Prior MI	23 (31.9) ^ ^,&^	19 (19.2)	3 (7.5)	0.04
Atrial fibrillation	4 (5.6) ^	7 (7.1) ^	16 (40)	0.02
Thyroid disease	19 (26.4) ^	23 (23.2) ^	1 (2.5)	0.04
Autoimmune disease	5 (6.9)	7 (7.1)	0	0.52
Peptic ulcer disease	12 (16.7)	13 (13.1)	10 (25)	0.19
Smoking	11 (15.3) ^	22 (22.2) ^	2 (5)	0.04
**Medications at discharge:**				
ASA	57 (79.2)	64 (64.5) ^	26 (65.0)	0.02
β-blocker	36 (50.0)	7 (7.1)*, ^	21 (52.5)	0.00
Calcium blocker	42 (58.3) ^	99 (100.0) ^	9 (22.5)	0.00
ACEI/ARB	51 (70.8)	61 (61.6)	29 (72.5)	0.56
Statins	65 (90.3)	77 (77.8)	35 (87.5)	0.23
Nitrates	9 (12.5)	32 (32.3) ^	2 (5.0)	0.03
Trimetazidine	23 (31.9) ^	22 (22.2) ^	8 (20.0)	0.01
VKA	4 (5.6) ^	7 (7.1) ^	16 (40)	0.02

*Nebivolol; ^p < 0.05 angina due to epicardial coronary spasm/microvascular spasm vs. AChT negative; ^&^p < 0.05 angina due to epicardial coronary spasm vs. microvascular spasm. ACEI, inhibitor of angiotensin convertase enzyme; AChT, provocative test with acetylcholine; ARB, angiotensin receptor blocker; ASA, acetylsalicylic acid; MI, myocardial infarction; VKA, vitamin K antagonist.

**FIGURE 2:**
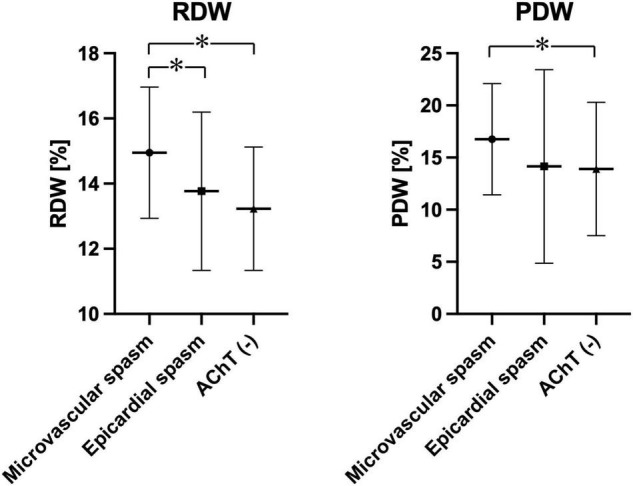
Plots showing RDW and PDW in the study population depending on the AChT result. *p < 0.05.

Table 2 presents 20 complete blood count parameters in three groups. The following parameters were significantly higher in patients with coronary microvascular spasm than in patients from the other two groups, i.e., epicardial coronary spasm and negative AChT: white blood cell (WBC) count, RDW, PLT count, MPV, and PDW. Figure 2 shows plots for RDW and PDW in all three groups.

**TABLE 2:** Comparison of values of 20 complete blood count parameters between groups.

Parameter	Microvascular spasm N = 72	Epicardial coronary spasm N = 99	Negative AChT N = 40	P
WBC [x10^3^/μL]	9.11 ± 3.34^&,^ ^	8.52 ± 2.68	8.11 ± 3.22	0.04
RBC [x10^6^/μL]	4.55 ± 0.62	4.68 ± 0.54	4.43 ± 0.36	0.732
HGB [g/dL]	13.92 ± 1.83	14.08 ± 1.54	14.19 ± 1.21	0.652
HCT [%]	40.82 ± 4.54	41.75 ± 4.07	41.79 ± 5.04	0.783
MCV [fL]	88.45 ± 4.77	89.79 ± 5.03	90.01 ± 4.67	0.689
MCH [pg]	30.13 ± 4.37	30.66 ± 5.00	30.78 ± 3.63	0.723
MCHC [g/dL]	33.04 ± 1.09	33.74 ± 1.14	33.90 ± 1.25	0.451
RDW [%]	14.95 ± 2.01^&,^ ^	13.77 ± 2.43	13.23 ± 1.89	< 0.001
PLT [x10^3^/μL]	267 ± 89	231 ± 125	245 ± 145	0.003
MPV [fL]	11.92 ± 0.89 ^	10.88 ± 1.10	10.11 ± 0.78	< 0.001
PCT [%]	0.23 ± 0.08	0.18 ± 0.23	0.18 ± 0.09	0.122
PDW [%]	16.76 ± 5.33 ^	14.15 ± 9.28	13.91 ± 6.39	< 0.001
LYM [x10^3^/μL]	3.34 ± 1.95	3.19 ± 2.38	3.01 ± 2.55	0.09
MON [x10^3^/μL]	0.48 ± 0.11	0.43 ± 0.01	0.46 ± 0.12	0.442
NEU [x10^3^/μL]	5.05 ± 2.34	4.78 ± 2.02	4.44 ± 2.87	0.549
EOS [x10^3^/μL]	0.20 ± 0.08	0.11 ± 0.07	0.15 ± 0.02	0.244
BASO [x10^3^/μL]	0.04 ± 0.01	0.01 ± 0.01	0.05 ± 0.01	0.577
NLR	1.58 ± 0.29	1.49 ± 0.57	1.40 ± 0.34	0.06
PLR	79.94 ± 44.90	72.41 ± 35.62	81.3 ± 43.54	0.192

*^p < 0.05 angina due to epicardial coronary spasm/microvascular spasm vs AChT negative; ^&^p < 0.05 angina due to epicardial coronary spasm vs microvascular spasm. BASO, basophil count; EOS, eosinophil count; HCT, hematocrit; HGB, hemoglobin; LYM, lymphocyte count; MCH, mean cell hemoglobin; MCHC, mean cell hemoglobin concentration; MCV, mean cell volume; MON, monocyte count; MPV, mean platelet volume; NEU, neutrophil count; NLR, neutrophil-lymphocyte ratio; PCT, plateletcrit; PDW, platelet distribution width; PLR, platelet-lymphocyte ratio; PLT, platelet count; RBC, red blood cell count; RDW, red cell distribution width; WBC, white blood cell count.*

Next, we performed univariable and multivariable logistic regression analysis to identify ultimately independent predictors of coronary microvascular spasm diagnosis in AChT. In the multivariable logistic regression analysis, the independent risk factors were female sex [odds ratio (OR), 1.199, 95% CI 1.001–1.329, *p* = 0.04], PDW (OR, 2.891, 95% CI 1.672–3.932, *p* < 0.001), and RDW (OR, 1.567; 95% CI 1.382–1.987, *p* < 0.01, Table 3). No significant findings were shown in angina due to epicardial coronary spasm or negative AChT groups (data not shown).

**TABLE 3:** Independent predictors in multivariable logistic regression analysis and Cox regression analysis.

Variable	Multivariable analysis	
	OR (95% CI)	*P*
**Independent predictors of coronary microvascular spasm**		
Female sex	1.199 (1.001–1.329)	0.04
MPV	1.112 (0.988–1.342)	0.06
PDW	2.891 (1.672–3.932)	< 0.001
RDW	1.567 (1.382–1.987)	0.0043

	**HR (95% CI)**	**P**

**Independent predictors of MI/chest pain hospitalizations in coronary microvascular spasm group at 5 years**		
Female sex	1.433 (1.288–1.782)	0.03
MPV	1.101 (1.002–1.345)	0.04
PDW	2.923 (1.789–3.332)	< 0.001
RDW	1.732 (1.431–2.344)	0.003

*MPV, mean platelet volume; PDW, platelet distribution width; RDW, red cell distribution width.*

Furthermore, a ROC analysis was performed for PDW and RDW as markers in predicting coronary microvascular spasm. PDW showed the highest sensitivity (65%) and specificity (72%) at the cutoff value of 15.32% [area under the curve (AUC), 0.723; 95% confidence interval (CI) 0.64–0.83; *P* < 0.001], and RDW characterized the following parameters: sensitivity (61%) and specificity (69%) at the cut-off value of 14.12% (AUC 0.642; 95% CI 0.543–0.738; *P* < 0.001).

**FIGURE 3:**
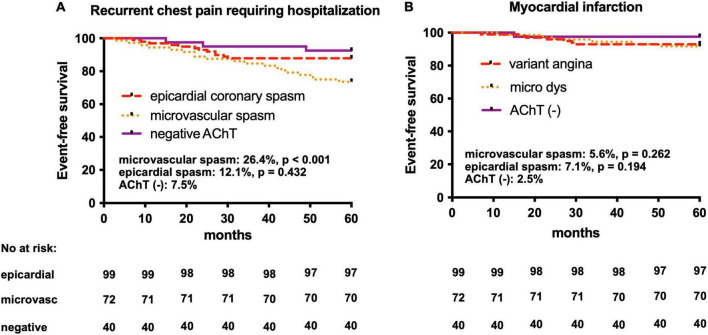
Kaplan–Meier curves in the AChPOL population. **(A)** Event-free survival from recurrent chest pain events requiring hospitalization at 5-year follow-up in three groups: epicardial coronary spasm, microvascular spasm and negative AChT **(B)** Event-free survival form myocardial infarction at 5-year follow-up in three groups: epicardial coronary spasm, microvascular spasm and negative AChT. AChT, provocative test with acetylcholine. Log-rank test: epicardial spasm vs. AChT negative groups and microvascular spasm vs. AChT negative groups.

At the 5-year follow-up (median, 55 months; range, 48–60 months), in the coronary microvascular spasm group, there were two non-cardiac deaths (2.8%), while six MIs (5.6%) and recurrent chest pain requiring hospitalization were observed in 19 patients (26.4%) (Figure 3). In all patients with MI control angiography was performed. In case of recurrent chest pain requiring hospitalization, control angiography was mainly performed after 18–24 months since the baseline AChT. Only in two patients (after 48 months and after 54 months) significant stenoses developed, and in one patient, after fractional flow reserve assessment, percutaneous coronary intervention with stent deployment was performed. In Table 4 we present data of clinical outcomes at 5 years in all three groups. Patients with microvascular spasm characterized the highest rate of recurrent chest pain leading to hospitalization, i.e., 26.4% vs. 12.1% in epicardial coronary spasm vs. 7.5% in AChT negative group (*p* = 0.02).

**TABLE 4:** 5-year clinical outcomes based on AChT.

Outcome N (%)	Total population *N* = 211	Microvascular spasm *N* = 72	Epicardial coronary spasm *N* = 99	Negative AChT *N* = 40	*P*
Recurrent chest pain requiring hospitalization	34 (16.1)	19 (26.4)	12 (12.1)	3 (7.5)	0.02
Myocardial infarction	14 (6.6)	6 (5.6)	7 (7.1)	1 (2.5)	0.23
Cardiac death	1 (0.5)	0	1 (1.0)	0	0.89
Death	4 (1.9)	2 (2.8)	2 (2.0)	0	0.67

*AChT, acetylcholine provocative test.*

In consequence, we performed Cox regression analysis to identify independent predictors of MI/chest pain hospitalization in the coronary microvascular spasm group: female sex (HR, 1.433, 95% CI 1.288–1.782, *p* = 0.03), MPV (HR, 1.101, 95% CI 1.002–1.345, *p* = 0.04), PDW (HR, 2.923, 95% CI 1.789–3.332, *p* < 0.001) and RDW (HR, 1.732; 95% CI 1.431–2.344, *p* = 0.003, Table 3).

## Discussion

To the best of our knowledge, our study is the first to investigate the predictive value of complete blood count indices in patients with coronary microvascular spasm. We showed that PDW and RDW were significantly associated with the diagnosis of coronary microvascular spasm in patients undergoing AChT as well as with poor prognosis in such patients at the 5-year follow-up.

The RDW is a measure of red blood cell volume variations (anisocytosis) and can be easily obtained during a routine complete blood count (24). In general, RDW is used in the differential diagnosis of anemia, especially that caused by deficiencies in mineral and vitamin levels, i.e., vitamin B_12_, folate, and iron. However, many reports have shown that elevated RDW level can be associated with CVDs and might be used as a predictor of high mortality in IHD patients (25), heart failure (26), and acute MI and also in the general population (27).

Although MPV was always considered a good predictor in cardiac patients, PDW currently is considered a more specific PLT reactivity factor (28). The PDW is a parallel measure to RDW because it shows PLT volume variations (PLTs vary in size and number of pseudopodia) (29). Elevated PDW indicates coagulation activation. PDW is valuable in predicting left ventricular dysfunction in ACS patients undergoing percutaneous coronary interventions (28). PDW has also been associated with the IHD severity in ACS patients (30).

Recently, Bekler et al. investigated that higher PDW (>17%) can be connected to the severity of CAD in patients with acute cardiac syndrome (30). According to the study, the higher the PDW, the higher also the Gensini score (a scoring system that determines the severity of CAD) (OR, 1.91; 95% CI, 1.27–2.88; *P* = 0.002). However, higher Gensini score also was associated with diabetes mellitus and MI (OR, 2.85; 95% CI, 1.91–4.25; *P* < 0.001 and OR, 2.67; 95% CI, 1.74–4.1; *P* < 0.001, respectively). Nevertheless, no correlation between PDW or MPV and the prevalence and severity of CAD (OR 0.99; 95% CI, 0.90–1.09; *P* = 0.87 and OR, 1.05; 95% CI, 0.95–1.16; *P* = 0.3; and adjusted OR, 0.97; 95% CI, 0.87–1.08; *P* = 0.63; respectively) was found in a large cohort study by De Luca et al. (31).

One key equivalent of coronary microcirculatory dysfunction is slow coronary flow (SCF) phenomenon, which can be recognized as a postponed distal vessel opacification without significant stenosis on coronary angiography. The mechanism of this angiographic phenomenon persists unclear, although a couple of concepts have been suggested, such as endothelial dysfunction, changes in blood rheological properties, inflammatory state, elevated uric acid concentration or conditions linked with an increased PLT volume. RDW and PDW were investigated to be predictors of the SCF phenomenon. In a retrospective study on 17,315 patients who underwent coronary angiography, Akpinar et al. found that elevated levels of those parameters may contribute to the microvascular blood flow resistance as the deformability of the cells is impaired (14). PDW also was associated with the presence and extent of SCF as reported by Seyyed-Mohammadzad et al. (*P* = 0.005) (32). Similar observations were proved in our study except for the role of Pct value.

Additionally, in our study, although the WBC count was within the normal limits in all groups, WBCs and neutrophils were observed in significantly higher numbers in the coronary microvascular spasm group compared to the other groups. These findings along with the increased RDW suggested that coronary microvascular spasm might be a subclinical inflammatory condition. However, other indices, such as NLR or PLR did not have a significant role. Altogether, the markers of increased inflammatory state which hamper the endothelial dysfunction as well as markers of procoagulant activity may predict ischemic events in coronary microcirculation. This may manifest benign as recurrent chest pain decreasing quality of life. However, it may lead to acute coronary syndromes or malignant ventricular arrhythmias.

### Limitations

The number of enrolled patients as well as the number of adverse events at follow-up were relatively low. Also, not enrolling consecutive patients could have been a source of bias. Moreover, high-sensitivity C-reactive protein or other inflammatory markers were not evaluated routinely in subjects undergoing AChT and was not included to verify the inflammatory status. Moreover, due to the limited population only chosen variables were used in the regression model.

## Conclusion

To the best of our knowledge, our study is the first to investigate the predictive value of complete blood count indices in patients with coronary microvascular spasm. PDW and RDW were significantly associated with the diagnosis of coronary microvascular spasm in patients undergoing AChT as well as with poor prognosis in such patients at 5-year follow-up.

## Data Availability Statement

The raw data supporting the conclusions of this article will be made available by the authors, without undue reservation.

## Ethics Statement

The studies involving human participants were reviewed and approved by Bioethics Committee at the Central Clinical Hospital of the Ministry of Interior and Administration. The patients/participants provided their written informed consent to participate in this study.

## Author Contributions

All authors planned and performed the study and took active role in manuscript preparation and final approval.

## Conflict of Interest

The authors declare that the research was conducted in the absence of any commercial or financial relationships that could be construed as a potential conflict of interest.

## Publisher’s Note

All claims expressed in this article are solely those of the authors and do not necessarily represent those of their affiliated organizations, or those of the publisher, the editors and the reviewers. Any product that may be evaluated in this article, or claim that may be made by its manufacturer, is not guaranteed or endorsed by the publisher.
